# Sea Surface Wind Speed Retrieval from Marine Radar Image Sequences Based on GLCM-Derived Texture Features

**DOI:** 10.3390/e27080877

**Published:** 2025-08-19

**Authors:** Hui Wang, Haiyang Qiu, Lei Wang, Jingxi Huang, Xingbo Ruan

**Affiliations:** 1 School of Low-Altitude Equipment and Intelligent Control, Guangzhou Maritime University, Guangzhou 510725, China; wanghui@gzmtu.edu.cn (H.W.); hxi664970@gmail.com (J.H.); ruanxingbo@gmail.com (X.R.); 2 State Key Laboratory of Information Engineering in Surveying, Mapping and Remote Sensing, Wuhan University, Wuhan 430072, China; lei.wang@whu.edu.cn

**Keywords:** X-band marine radar, wind speed, GLCM-derived features, energy value, entropy value

## Abstract

Sea surface wind speed is a key parameter in marine meteorology, navigation safety, and offshore engineering. Traditional marine radar wind speed retrieval algorithms often suffer from poor environmental adaptability and limited applicability across different radar systems, while existing empirical models face challenges in accuracy and generalization. To address these issues, this study proposes a novel wind speed retrieval method based on X-band marine radar image sequences and texture features derived from the Gray-Level Co-occurrence Matrix (GLCM). A three-stage preprocessing pipeline—comprising noise suppression, geometric correction, and interpolation—is employed to extract small-scale wind streaks that reflect wind field characteristics, ensuring high-quality image data. Two key GLCM texture features of wind streaks, energy and entropy, are identified, and their stable values are used to construct a segmented dual-parameter wind speed model with a division at 10 m/s. Experimental results show that both energy- and entropy-based models outperform traditional empirical models, reducing mean errors by approximately 49.3% and 16.7%, respectively. The energy stable model achieves the best overall performance with a correlation coefficient of 0.89, while the entropy stable model demonstrates superior performance at low wind speeds. The complementary nature of the two models enhances robustness under varying conditions, providing a more accurate and efficient solution for sea surface wind speed retrieval.

## 1. Introduction

The ocean wind field plays a crucial part in both onshore and offshore activities and in the research of the marine environment [1]. Among these, the sea surface wind speed is one of the essential parameters for offshore meteorological forecasting, ship navigation, and marine resource exploitation. Specifically, it offers a scientific foundation for the takeoff and landing of carrier-based aircraft, the assessment of offshore wind power and wind energy, as well as the prediction of safe navigation speeds [2]. Currently, there are primarily two approaches for detecting sea surface wind speed information, namely on-site measurement and remote sensing retrieval [3]. For on-site measurement, anemometers are usually installed on buoys, ships, or shore-based to directly obtain wind speed. However, those installed on buoys lack continuity in time and space, while those installed on ships or shore-based are susceptible to turbulence effects caused by ship structures, buildings, or shore-based obstacles, resulting in significant errors and failing to meet practical applications [4]. Remote sensors such as scatterometers, synthetic aperture radar (SAR) carried by aircraft or satellites, and satellite altimeters have been extensively utilized to retrieve wind field information over large areas [5]. Owing to the high spatiotemporal resolution and low additional cost of X-band marine radar [6], it is widely employed to retrieve ocean wave heights [7,8], currents [9], rainfall detection [10,11], and sea surface wind field [12,13].

A number of theoretical analyses and experiments have been carried out to retrieve wind speed from the radar images. One approach is the machine learning (ML) method. Dankert et al. [14] set up a BP neural network model by utilizing the mean crosswind echo intensity of radar images, along with measured wind field information. Sea state information and atmospheric factors were then introduced as input parameters into the neural network, and the model’s applicability under different sea conditions was enhanced. The K-means clustering algorithm was applied by Wang et al. [15] to remove outliers from the input network data, and the processed data was fed into an improved RBF neural network, thus improving the accuracy of the wind speed model. Even though the application of neural networks is not influenced by imaging mechanisms, a substantial amount of training data is required to guarantee the generalization ability and accuracy of the model. Later, more advanced ML methods were proposed to retrieve wind speed, such as support vector regression (SVR)-based [16], convolutional NN (CNN)-based [17], and wavelet scattering transform CNN (WSTCNN) [18], which effectively improved model accuracy and robustness under complex environmental conditions. However, when it comes to different marine environments and various types of navigation radars, it becomes necessary to collect new data and retrain the model. This significantly restricts the applicability and portability of these methods.

An alternative approach is to acquire sea surface wind speed information directly by relying on an empirical function model. Hatten et al. [19] found that there is a strong correlation between the background noise of marine radar sea clutter images and the information of sea surface wind field information, and proved that the total variance of the spectral background noise of radar cross section (RCS) can be applied to extract the information of sea surface wind speed. Izquierdo and Soares [20] proposed a linear relationship model between the spectral background noise of sea clutter images and the sea surface wind speed and established the first empirical function model for sea surface wind speed. Lund et al. [21] proposed establishing a functional relationship between the mean value of the range image of the navigation radar (Furuno FAR2117BB) and the sea surface wind speed, and verified the feasibility of directly applying the radar echo intensity to extract the information of sea surface wind speed. Vicen-Bueno et al. [22] proposed using the echo intensity level selection method of the range of a single radar image to establish an empirical model between the echo intensity level and the sea surface wind speed and improve the accuracy of directly applying radar images to extract sea surface wind speed information. However, further discussion is still needed regarding the universality of this model. Liu et al. [23] proposed an approach that uses a hyperbolic fitting technique to improve the wind speed model in relation to the radar echo intensity level for both Decca and Furuno radars, thereby improving the radar universality of this method. Chen and He et al. [24] set up an empirical function that relates sea surface wind speed, radar echo intensity, and significant wave height. This enhanced the model’s applicability in marine environments. Lu and Yang et al. [25] established a functional relationship between significant wave height and wind speed under wind wave conditions. Sea surface wind speed information can be retrieved based on this model. However, this method exhibits a relatively large error when the swell is dominant. Liu and Huang et al. [26] developed an empirical mode decomposition function model of radar cross section (RCS) and the sea surface wind field. On the premise that the radar echo intensity is known, information about wind speed and wind direction can be retrieved. Existing function models predominantly establish relationships between the echo intensity of a single radar image and the measured wind speed. However, the correlation between the echo intensity of radar images and wind speed varies across different distance ranges and azimuth angles. Additionally, current methods are incapable of directly mitigating the impact of noise and interference present in radar images. These factors jointly contribute to a reduction in retrieval accuracy and limit the engineering application potential. Consequently, there remains a pressing need to explore a more precise and efficient approach for wind speed estimation.

The gray-level co-occurrence matrix (GLCM) analyzes radar texture image features by calculating the probability of echo intensity co-occurrence at specific directions and distances. The derived texture features, such as Energy and Entropy, directly reflect the complexity of the image [27]. GLCMs are widely used in image classification, industrial inspection, and computer vision applications [28].

In airborne synthetic aperture radar (SAR) images, large-scale static wind streaks (ranging from 1 to 2 km) parallel to the sea surface wind direction are formed due to thermodynamic rolls in the atmospheric boundary layer. These streaks enable the extraction of wind direction parameters [29,30]. Similarly, in X-band marine radar images, small-scale wind streaks (ranging from 200 to 500 m) aligned with the surface wind direction emerge due to sea surface wind shear stress [31]. These streaks persist longer than surface waves [32] and provide a basis for wind direction retrieval in both the frequency [33] and spatial domains [34,35,36,37]. However, while the numerical texture parameters derived from these streaks have been effectively used for wind direction retrieval, their potential for retrieving sea surface wind speed information remains unexplored. This gap highlights an important opportunity for further research.

This study validates the correlation between GLCM-derived numerical texture features, such as entropy and energy, and measured sea surface wind speeds using small-scale wind streak images from marine radar sequences. Experimental results demonstrate a clear linear relationship between the stable values of entropy and energy and wind speeds in the range of 0 to 20 m/s. Based on these findings, a new wind speed retrieval model is developed and tested in regions of radar images with the highest wind speed sensitivity. Robustness testing further confirms that the model maintains reliable performance even when applied to radar image sequences with noise and interference, including those without precise calibration, showcasing its practical applicability in real-world scenarios.

The remainder of this paper is structured as follows. In Section 2, a detailed description of data pre-processing is provided, which involves two main aspects. Firstly, it includes the extraction of small-scale wind streaks. Secondly, an in-depth investigation is carried out regarding the characteristics exhibited by these streaks under varying wind speed conditions. Section 3 presents the methodology for an ocean wind speed retrieval algorithm based on marine radar image sequences and showcases the simulated wind speed model within the context of GLCMs. Section 4 presents an analysis of the wind speed results derived from X-band marine radar data using different models and further explores the influence of various sea conditions, particularly significant wave height, on the energy and entropy stable model. Finally, Section 5 offers a summary and a conclusion.

## 2. Retrieving Wind Speed Using the Empirical Function Model

For HH-polarized marine radar images, the normalized radar cross section (NRCS) is highly dependent on sea surface wind speed and radar incidence angle. Studies have shown that these three factors obey a Geophysical Model Function (GMF) [19], demonstrating the feasibility of retrieving wind speed from marine radar under known wind direction conditions. Extensive experiments indicate a positive correlation between the average NRCS of radar images and wind speed, and that NRCS and wind speed follow a cubic polynomial relationship [21]. However, actual ocean wave dynamics are complex. Generally, for wind seas, higher wind speeds correspond to greater significant wave heights; for swells, the significant wave height should theoretically increase with wind speed, but sometimes a very high wind speed is accompanied by a very low wave height. Differences in wind fetch and duration across regions mean the wind speed–wave height relationship varies greatly by location, and distinguishing wind sea from swell adds further complexity. Reference [25] used Wilson’s wind speed–wave height model to compensate and correct the cubic polynomial, proposing an improved empirical function model:

$$\sigma_{wSpd}=\frac{1}{2\pi }\int_{0}^{2\pi }\left(\alpha_{0}+\alpha_{1}\cos{\left(0.5(\theta -\alpha_{2})\right)}^{2}\right)d\theta$$

(1) $$u_{wind}=p(m\sigma_{wSpd}+n){\left(\frac{gH_{sw}}{\gamma }\right)}^{q}$$

In this model, θ is the angle between the wind direction and the antenna’s look direction. $\sigma_{wSpd}$ is the average NRCS obtained from the radar image. $H_{sw}$ denotes the significant wave height, g is the gravitational acceleration, and m, n, γ, p, and q are empirical parameters. Compared to the cubic polynomial model, this model offers some improvement in environmental applicability and achieves slightly higher retrieval accuracy at moderate wind speeds; however, it still determines model parameters solely from the mean echo intensity of the initial radar image without analyzing characteristics of the wind field imaging mechanism. Therefore, it has the following problems:

① Parameter inaccuracy: The wind field imaging mechanism is complex and influenced by many factors (e.g., atmospheric refraction, scattering characteristics, the geometry between the radar and the wind field). Relying only on mean echo intensity cannot fully reflect the effects of these factors, which may lead to model parameters deviating from actual wind field conditions and failing to capture the spatiotemporal variations and fine details of the wind field.
② Poor model adaptability: Different wind field environments (such as typhoon winds or localized strong convective winds) have unique characteristics. Without considering the wind field imaging mechanism, the chosen model parameters may only apply to radar images under specific conditions. For other types of wind fields or environments, the model’s adaptability and generalization are very poor, making accurate wind field retrieval difficult.
③ Large retrieval error: Because the model parameters may be inaccurate and not universally applicable, using this model for wind field retrieval can produce large errors. It might not accurately capture key information such as wind speed and direction, and assessments of wind field intensity, extent, and trends can be biased. This impairs scientific understanding of the wind field and its practical applications.
④ Neglect of important information: The wind field imaging mechanism contains important information related to wind characteristics (e.g., Doppler shift, polarization). Focusing only on mean echo intensity ignores this additional information, leading to an incomplete understanding of the wind field. This prevents deeper exploration of the wind field’s underlying physical processes and patterns, limiting more accurate analysis and research.

## 3. The GLCM-Derived Texture Features Wind Speed Estimation Model

**Figure 1:**
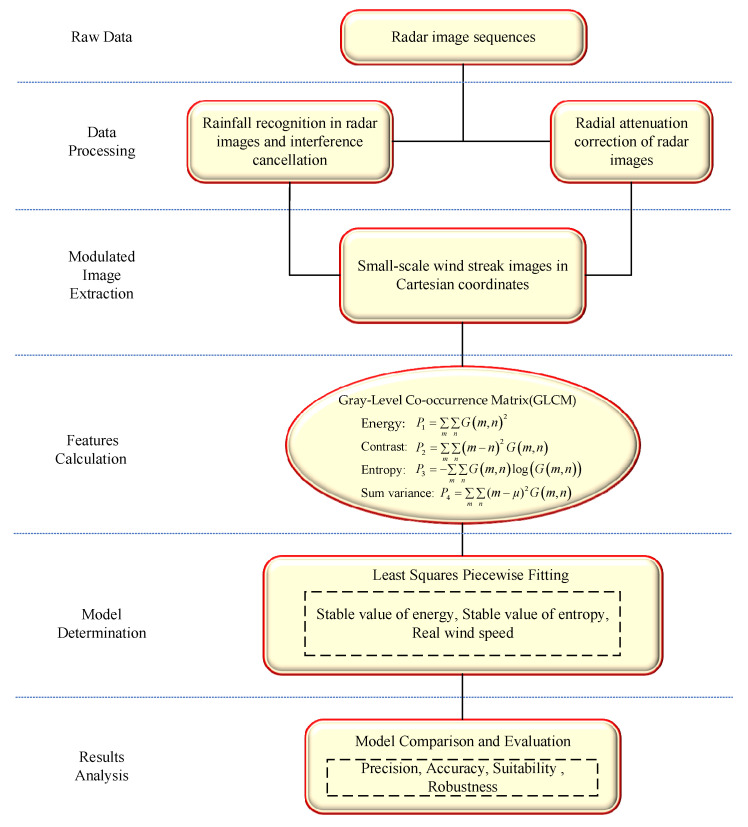
Flowchart of wind speed model design.

The wind speed function model developed in this paper is based on the imaging characteristics of the sea surface wind field observed by marine radar, effectively addressing the issues present in the above Empirical Function Model. The implementation flowchart is shown in Figure 1. In the first step, small-scale wind streaks in Cartesian coordinates are extracted from preprocessed images, which involve operations such as noise suppression, image correction, and image interpolation. Subsequently, the characteristic relationship between these wind streaks and the sea surface wind speed is determined. The subsequent emphasis is placed on investigating the correlation between the characteristics of small-scale wind streaks obtained from GLCM and the measured wind speed. This exploration leads to the determination of crucial feature values, specifically energy and entropy, which are found to be associated with wind speed. To address the issue of data differences affecting the modeling process, the stable values of applied energy and entropy are employed to represent the characteristics of the global data for model establishment. Eventually, two empirical function models are developed to describe the relationship between the stable values of energy and entropy and the segmented reference wind speed.

### 3.1. Small-Scale Wind Streaks Extraction

**Figure 2:**
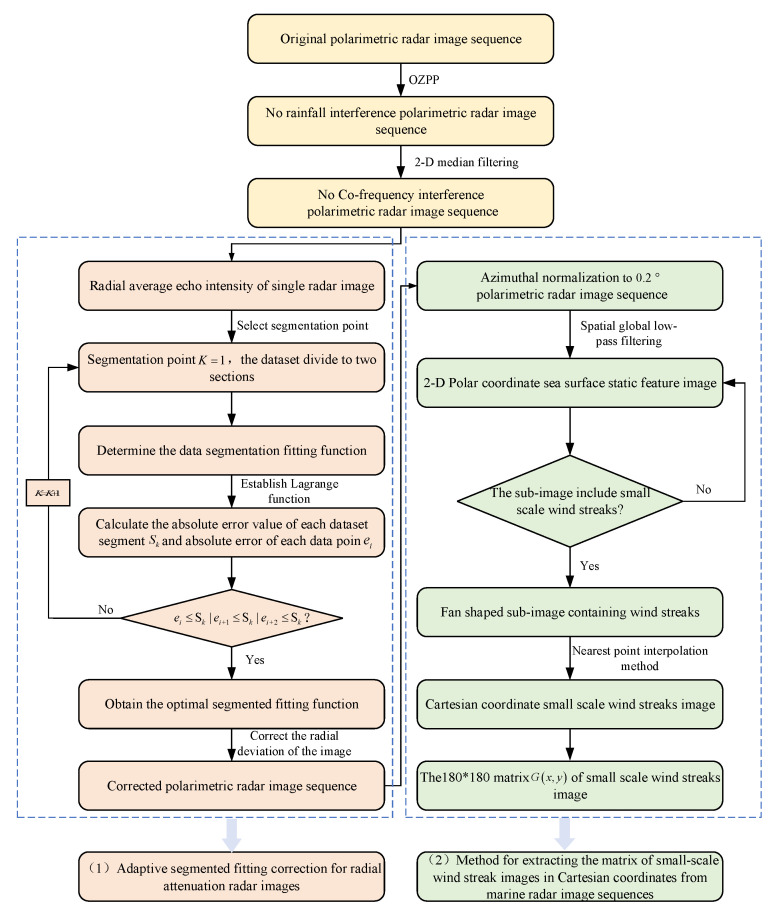
The pre-processing procedure of the radar image sequence.

For the purpose of extracting the image with small-scale wind streaks from the X-band marine radar image sequence, which is required for the calculation of features derived from the GLCM, the pre-processing procedure of the radar image sequence is illustrated in Figure 2.

The marine radar described in this paper operates with HH polarization. The antenna is positioned at a height of 40 m, and its horizontal beam width is less than 1.3°. It surveys the surrounding environment in a rotational scanning mode. The observation time for a single image is 2.5 s. Each radar image sequence consists of 32 images, which signifies a duration of approximately 80 s. Based on the antenna height and beam width, the grazing angle at which the radar electromagnetic wave is projected onto the sea surface is approximately within the range from 0.5° to 1.3°. It encompasses a sea surface area within a radius of approximately 4000 m and has a range resolution of around 7.5 m.

**Figure 3:**
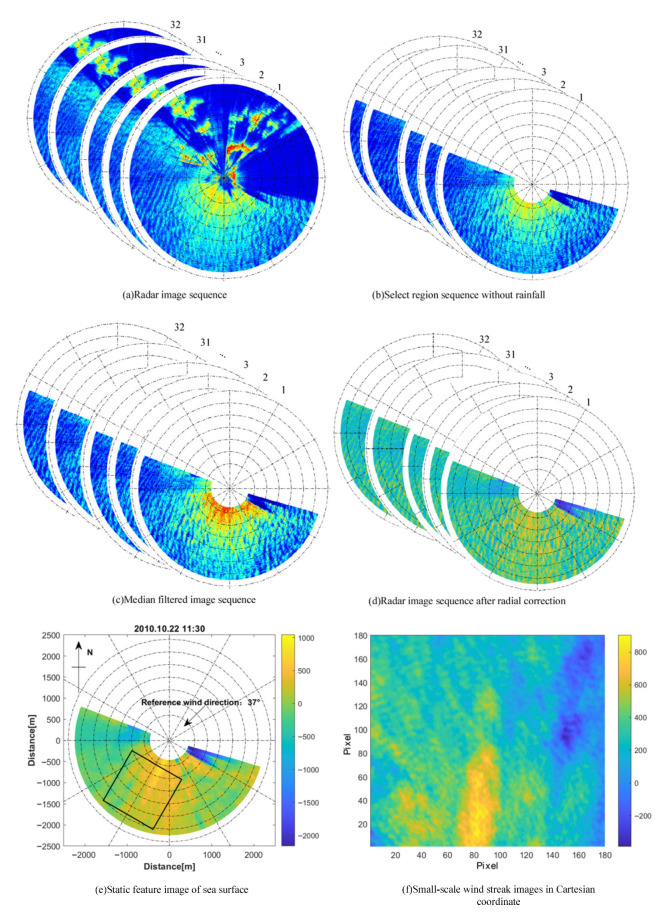
Pre-processing results of the radar image sequence (**a**) A radar image sequence (**b**) Select the region sequence without occlusion and rainfall (**c**) Median filtered image sequence (**d**) Radar image sequence after radial correction (**e**) Static feature image of sea surface (**f**) Small-scale wind streak image in Cartesian coordinate.

Figure 3a shows the radar image sequence recorded at 11:30 on 22 October 2010. In the area proximity to the radar antenna, the echo signal is prone to oversaturation. Conversely, at great distances, the echo signal is weakened and submerged in the background noise. In order to obtain ideal observation data, the intermediate echo area (600–2100 m from the radar) was selected. The portion of the radar image ranging from 106° in the counterclockwise direction to −69° was preserved, whereas the part beyond this range was directly removed. This was performed to eliminate the interference caused by shore-based fixed targets and to improve the capability for applying sea clutter wind speed modeling.

Some of the collected radar images are affected by rain, leading to blurred parts and the appearance of messy bright spots or speckles, especially in the high- and low-frequency parts of the radar image spectrum, which contain information about sea waves and wind fields. To accurately extract information about ocean waves and sea-surface wind fields, efficient rainfall suppression algorithms and signal-processing techniques are needed to separate or filter out rainfall noise and ensure the reliability of radar remote sensing data. In this study, the OZPP (occlusion area zero-pixel percentage) rainfall recognition method in [32] is employed to directly separate the rainfall images and preserve the image sequences without rainfall interference. Figure 3b presents the image sequence of the selected area at 11:30 on 22 October 2010, free from rainfall interference. At this moment, the reference wind direction is 37°, the wind speed is 17.2 m/s, the wave height is 2.64 m, and the wave direction is 110°. Interference originating from other marine radars gives rise to radial noise lines within radar images, which are manifested as dense radial pixel lines. Additionally, interference from targets such as ships leads to the emergence of bright spots in the radar images. In this study, a 3×3 template 2-D median filter is employed to process the images, and the processing results are presented in Figure 3c. Although the interference cannot be eradicated completely, the retrieval capability of the image is essentially preserved.

The small-scale wind streaks in marine radar images exhibit a radial attenuation characteristic similar to that of the radar signal. As the distance from the radar center increases, these wind streaks tend to become increasingly blurred. When the radial distance exceeds 1500 m, they are nearly indistinguishable and submerged in the attenuated background noise. In this study, the adaptive segmented fitting correction method for radially attenuated radar images proposed in [32] was applied. This algorithm performs refined corrections to the radar echo intensity of different distance segments based on the characteristics of the actual observation data, maximizing the preservation of small-scale wind streak features at long distances; thus, it improves the accuracy and reliability of wind speed retrieval. The corrected radar image sequence is shown in Figure 3d.

Since the radar antenna employs a rotational periodic scanning mode, the sampling angle interval is approximately 0.1°, but the covered angle is larger than this value. Therefore, a fixed angle of 0.2° is adopted for sampling. Consequently, there are 1800 lines for a 360° cycle, with 600 points on each line to reconstruct the single-polarized radar image. The marine radar image sequence after amplitude normalization is subjected to superposition and averaging processing along the time axis (with one cycle being approximately 80 s). Eventually, a static feature image of the sea surface, mainly characterized by small-scale wind streaks, is obtained, as shown in Figure 3e.

Research [38] shows that the mean value of the single-radar radial echo intensity is in line with the geophysical model function (GMF). According to the GMF model, it is evident that the normalized radar cross section (NRCS) exhibits an exponential function relationship with the sea wind speed and has a harmonic function relationship with the angle between the electromagnetic wave and the wind direction. The larger the wind speed is, the larger the NRCS will be, and the peak value of NRCS will appear when the wind direction is upwind (180° is upwind, 0° is downwind, 90° is crosswind). Under these conditions, a relatively bright area appears on the radar image. Conversely, the radar image is darker. This also shows the presence of bright and dark areas formed by wind field modulation in marine radar images, that is, the wind streak region.

To effectively extract the small-scale wind streak region, the upwind direction is used as the central axis of symmetry for selecting the feature region. The selected axis of the image region is 211° relative to the north under the polar coordinate image. A total of 180×180 pixel points are selected in the polar-coordinate image. One radar pixel is 7.5 m, so the selected area size is 1350×1350 m, ensuring that small-scale wind streaks are covered in the selected area, as shown in the black rectangle in Figure 3e. In order to calculate GLCM-derived features more accurately, the black rectangle image in Figure 3e, which is presented in polar coordinates, is first interpolated using a nearest-neighbor method. After interpolation, it is converted into a Cartesian coordinate image, as the GLCM calculation is more appropriate in the Cartesian coordinate system. Interpolation can fill in the blank points or sparse regions in the original image data to generate continuous and intact small-scale wind streak images crucial for retrieving the wind speed.

The position of pixels in polar coordinates is shown in the black rectangle in Figure 3e, and the coordinate of the Cartesian coordinate system needs to be established to calculate the feature values of the streaks. The pixel value of the point in polar coordinates $\left(r,\theta \right)$ with the nearest distance is determined as the pixel value of the Cartesian coordinates $\left(x,y\right)$, and the relationship between the two coordinates is as follows:

$$\left\{\begin{array}{l} x=r*\cos\theta \\ y=r*\sin\theta \end{array}\right.$$

(2) $$\left\{\begin{array}{l} r=round\left(sqrt\left(x^{2}+y^{2}\right)\right)\\ \theta =round\left(rem\left(a\tan\left(y,x\right)+2\pi ,2\pi \right)\right) \end{array}\right.$$

where $round\left(•\right)$ represent a modulo function, $rem\left(•\right)$ is a nearest-point rounding function, and $a\tan\left(•\right)$ is the inverse tangent function. Finally, the small-scale wind streak image in the Cartesian coordinate system is obtained, as illustrated in Figure 3f. The yellow bright streak region represents the small-scale wind streaks generated by the modulation of the sea surface wind field.

### 3.2. Streak Characteristics of Different Wind Speeds

**Figure 4:**
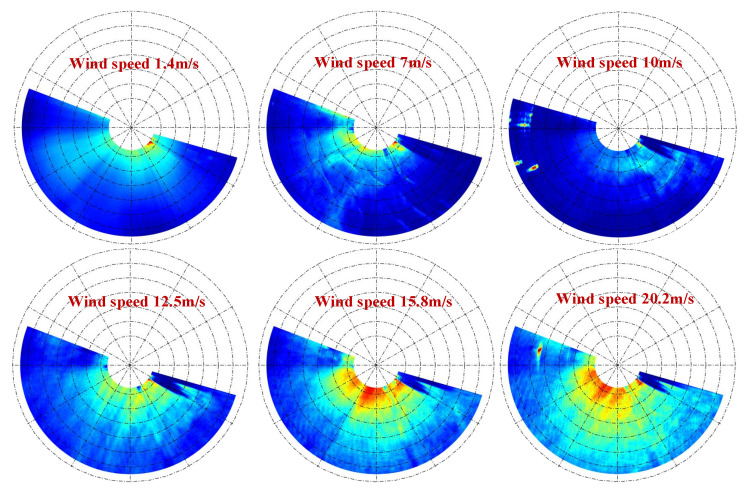
Small-scale wind streak images under different wind speeds.

Based on the hydrodynamic coupling between sea surface microscale waves and wind speed, the statistical characteristics of wind streaks are directly related to sea surface roughness, which in turn is modulated by local wind speed. This relationship was validated in [14] through Bragg scattering theory and empirical models. In order to examine the relationship between small-scale wind streaks and wind speed, the small-scale wind streaks in polar coordinates, which were obtained through the preprocessing process described in Section 3.1 under different wind speed conditions, were selected for comparative analysis, as shown in Figure 4. In Figure 4, the yellow highlighted area represents the small-scale wind streak image. It is observable that as the wind speed progressively rises, the small-scale wind streaks become more distinct and the contrast is heightened. However, when the wind speed is lower than 10 m/s, the small-scale wind streaks are extremely faint. The disturbance of the wind on the sea surface is small when the wind speeds are relatively low, resulting in relatively low sea-surface roughness [14]. Marine radar reflects the changes in sea surface roughness during sea surface observation; therefore, when the sea surface wind speed is below 10 m/s, the small-scale wind streaks that are formed are rather faint. Correspondingly, in the image, they appear to have low contrast and lack obvious texture structure. When the wind speed exceeds 10 m/s, all the small-scale wind streaks display prominent features and high contrast.

### 3.3. Calculating the Features of Small-Scale Wind Streaks Based on GLCM

Small-scale wind streaks are features of marine radar imaging modulated by the sea surface wind field, which contain directionality, periodicity, and texture of the sea surface wind field. By calculating the asymmetrical GLCM of the wind streak, the joint probability distribution of the gray level of a pair of pixels for any given relative position can be obtained. Compared with the traditional GLCM, the asymmetrical GLCM can flexibly select different relative pixel positions for calculation, so that it can capture the texture information in the image more meticulously and cover a broader range of relative pixel position relationships.

The Cartesian coordinate system shows the variation range of the pixel intensity of $f\left(x,y\right)$ with respect to $\left(x,y\right)$. After performing grayscale conversion and normalization processing on the wind streak image, we assume that the grayscale level range of the image is from 0 to N−1 and the GLCM is an N×N matrix. In order to estimate the GLCM, only the correlation of a single pixel in the streak image is taken into consideration. In Cartesian coordinates, the relative positions of pixel pairs are defined by distance d and direction θ, and are represented as $\left(d,\theta \right)$. For a GLCM of relative position $\left(d,\theta \right)$ coinciding with the pixels, its matrix element (m,n) can be calculated by counting the pixel pairs as follows [29]:

(3) $$G\left(m,n;d,\theta \right)=card\left(\begin{array}{l} \left(x,y\right)|f\left(x,y\right)=m-1,\\ f\left(x+d\cos\theta ,y+d\sin\theta \right)=n-1,\\ \left(x,y\right)\in Ρ,\left(x+d\cos\theta ,y+d\sin\theta \right)\in Ρ \end{array}\right)/Q\left(d,\theta \right)$$

where $card\left(•\right)$ represents a counting function, with its output being the number of elements within the set. The distance increment is set at 1 pixel, and the angle increment is 0.1°. The pixel set of the image in the rectangular coordinate system is Ρ. $Q\left(d,\theta \right)$ is the normalization factor, representing the total number of pixel pairs that satisfy the relative position $\left(d,\theta \right)$. With the pixel spacing as the distance unit, it can be expressed as follows:

(4) $$Q\left(d,\theta \right)=card\left\{\left(x,y\right)|\left(x,y\right)\in Ρ,\left(x+d\cos\theta ,y+d\sin\theta \right)\in Ρ\right\}$$

A group of GLCM-based parameters can be derived from GLCM [39]. Four key parameters directly reflecting the characteristics of the wind streaks can be identified: energy $P_{1}$, contrast $P_{2}$, entropy $P_{3}$, and sum variance $P_{4}$. Based on this, the relationship between the streak features and the sea surface wind speed can be quantitatively analyzed. The four parameters are expressed as follows:

(5) $$\mathrm{Energy}:\;P_{1}=\sum_{m}\sum_{n}G{\left(m,n\right)}^{2}$$

(6) $$\mathrm{Contrast}:\;P_{2}=\sum_{m}\sum_{n}{\left(m-n\right)}^{2}G\left(m,n\right)$$

(7) $$\mathrm{Entropy}:\;P_{3}=-\sum_{m}\sum_{n}G\left(m,n\right)\log\left(G\left(m,n\right)\right)$$

(8) $$\mathrm{Sum}\;\mathrm{variance}:\;P_{4}=\sum_{m}\sum_{n}{\left(m-\mu \right)}^{2}G\left(m,n\right)$$

In Equation (8), μ is the mean of the matrix G. When the energy $P_{1}$ and sum variance $P_{4}$ are at a high level, the small-scale wind streaks present in the sea surface static feature image show more regularity and are more stable. If contrast is high and entropy is low, it usually means that there are obvious and high-contrast texture features in the image. In this case, subject to wind force, the boundaries of wind streaks in a single direction are relatively clear.

### 3.4. Analysis of the Relationship Between GLCM-Derived Features and Wind Speed

**Figure 5:**
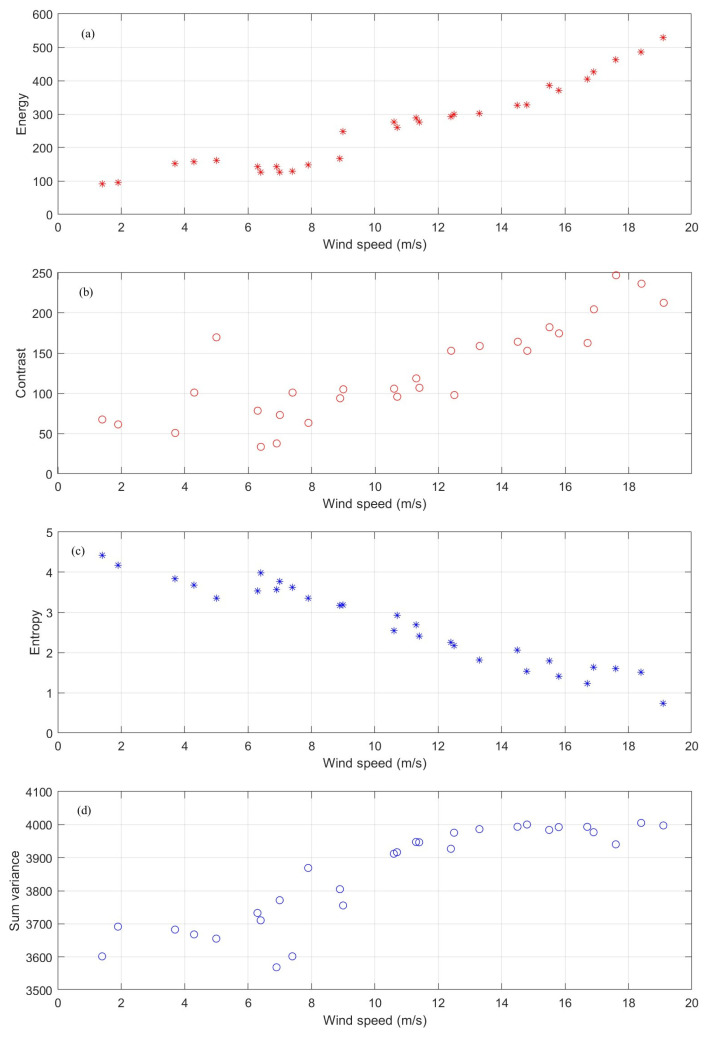
GLCM-derived features that change with different wind speeds. (**a**) Energy. (**b**) Contrast. (**c**) Entropy. (**d**) Sum variance.

To analyze and validate the relationship between the GLCM-derived parameters and the sea surface wind speed, 25 sets of marine radar image sequences under different wind speeds ranging from 3 to 20 m/s were selected. The aforementioned four parameters—energy, contrast, entropy, and sum variance—were computed, as illustrated in Figure 5. Figure 5 shows that when the wind speed increases, energy, contrast, and sum variance display a progressively increasing tendency, whereas entropy decreases gradually in tandem with the decrease in wind speed; likewise, with the increase in wind speed, the energy, contrast, and sum variance show a gradually increasing trend, while the entropy declines steadily. Consequently, when the wind speed surges (i.e., at higher wind speeds), the small-scale wind streaks become more orderly and stable, and their boundaries become more distinct, corresponding to the trends observed in the wind streak images shown in Figure 4.

Figure 5 also shows that all four parameters are rather unstable and undergo significant fluctuations when the wind speed is lower than 10 m/s. However, energy and entropy still retain a relatively consistent relationship with the sea surface wind speed. Due to the limited amount of sampling data, it is impossible to directly establish a wind speed model, and further research is required to make a determination.

In Figure 5, the trends for energy, contrast, and sum variance are all directly proportional to the wind speed. In subsequent research, energy and entropy can be selected to construct a wind speed model, which will significantly simplify the model determination process.

### 3.5. Wind Speed Estimation Model

Owing to the extensive volume of sampled data and the presence of outliers with significant variability, accurately determining the trend of the sea surface wind speed model poses a considerable challenge. To identify stable energy and entropy values, a wind speed model is established by leveraging stable value features that reflect the global data properties. The GLCM of small-scale wind streaks is computed for each radar image sequence, and energy and entropy features are derived from Equations (5) and (7), as described in Section 3. In order to mitigate the impact of unstable data, the mean and variance of the energy and entropy values obtained from every seven consecutive radar sequences are calculated.

**Figure 6:**
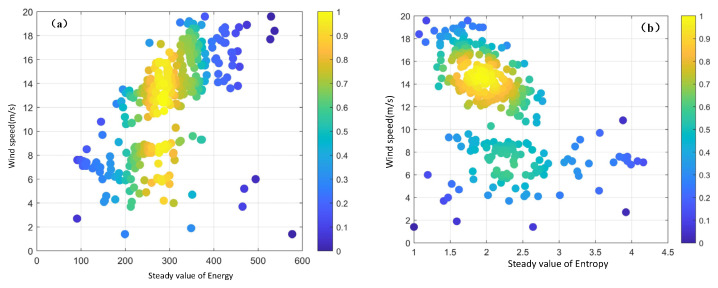
Variation of stable values of energy and entropy with wind speed. (**a**) Stable value of energy. (**b**) Stable value of entropy.

Each group of radar image sequences lasts approximately 80 s, which is roughly equivalent to a 10 min sequence for calculation. The standard deviation and mean are obtained and then evaluated using the probabilistic statistical method. If the standard deviation of the energy and entropy values from seven consecutive radar sequences is less than 1% of their respective means, it indicates that the eigenvalue data during this period is highly stable and concentrated. This implies that energy and entropy have reached a stable state. Subsequently, the means of energy and entropy eigenvalues of these seven radar sequences are taken as the stable feature values. Through the calculation of 2184 sets of marine radar image sequences, 312 sets of stable values of the energy and entropy features of the GLCM are ultimately obtained. The correlation with the measured sea-surface wind speed is illustrated in Figure 6.

The relationship between the energy stability value and the measured wind speed is depicted in Figure 6a, while the relationship between the entropy stability value and the sea surface wind speed is shown in Figure 6b. It can be observed that the relationships between the stable energy value, the stable entropy value, and the wind speed are piecewise-function relationships, and a wind speed of 10 m/s serves as a clear demarcation point. In order to model the sea surface wind speed more precisely, a least-squares method is employed to fit the stable energy value and stable entropy value data separately for wind speeds below and above 10 m/s.

**Figure 7:**
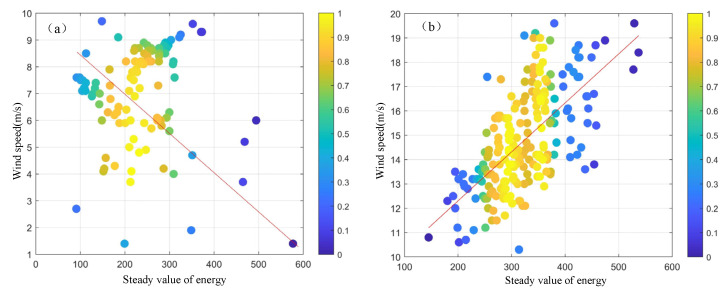
The relationship between the stable value of energy and the wind speed. (**a**) When the wind speed is below 10 m/s. (**b**) When the wind speed is above 10 m/s.

The relationship between the energy stable value and the sea surface wind speed is depicted in Figure 7. In Figure 7a, the relationship between the stable energy value and the measured wind speed at wind speeds below 10 m/s is shown, while in Figure 7b, the relationship between the energy stable value and the sea surface wind speed at wind speeds above 10 m/s is illustrated. The least-squares method was used to perform piecewise fitting for each dataset, and the relationship model is obtained as follows:

(9) $$y_{s}=\left\{\begin{array}{l} -0.001x_{e}+8.7,\;y_{s}\leq 10\\ 0.02x_{e}+8.3,\;y_{s}>10 \end{array}\right.$$

where $x_{e}$ represents the energy stable value and $y_{s}$ is the measured wind speed. The fitting curve of the final energy stable value and wind speed is shown as the red line in Figure 7.

**Figure 8:**
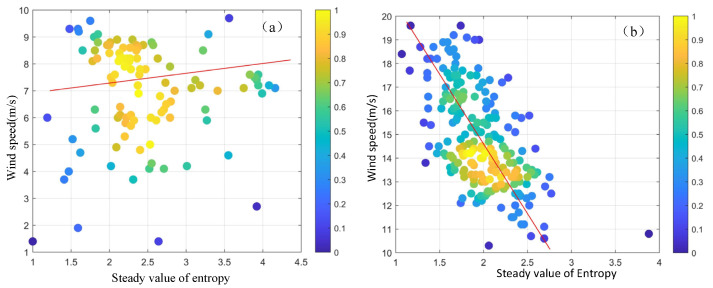
The relationship between the stable value of entropy and the wind speed. (**a**) When the wind speed is below 10 m/s. (**b**) When the wind speed is above 10 m/s.

The relationship between the entropy stable value and the measured wind speed is illustrated in Figure 8. Figure 8a presents the relationship when the measured wind speed is below 10 m/s, while Figure 8b is for wind speeds above 10 m/s. Finally, the entropy stable value and measured wind speed function model is obtained as follows:

(10) $$y_{s}=\left\{\begin{array}{l} 0.32x_{tr}+7.5,\;y_{s}\leq 10\\ -4.3x_{tr}+23,\;y_{s}>10 \end{array}\right.$$

where $x_{tr}$ is the entropy stable value. The fitting curve of the final entropy stable value and the sea surface wind speed is shown as the red line in Figure 8.

It was found that the correlations between the energy and entropy stable values of the model and the sea surface wind speed above 10 m/s are consistent with those in Section 3.2. However, the characteristics are reversed when the wind speed is below 10 m/s. At such low wind speeds, the wind’s disturbance on the sea surface is relatively minor, resulting in extremely weak wind streaks. This leads to low contrast and a lack of texture structures in the radar image, causing the stable feature values calculated by the GLCM to be scattered. Moreover, the differences in feature values among various image sequences are not significant, and the correlation with the sea surface wind speed is rather weak. Given the relatively small number of samples with a wind speed under 10 m/s encompassed in the current experimental radar data, the fitting performance of the model within the low wind speed range is adversely affected. To optimize the model for the low wind speed area, additional sample data are needed.

## 4. Validation and Testing

### 4.1. Data Overview

**Figure 9:**
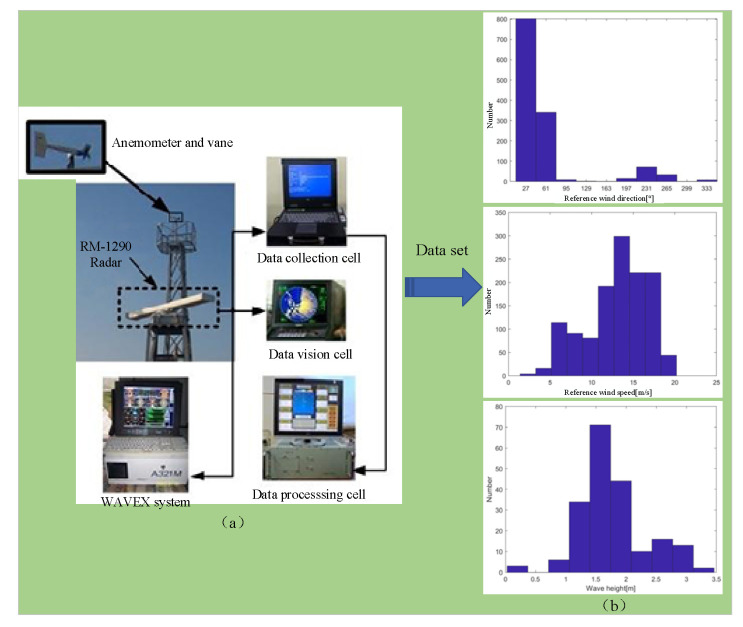
Data sources and distribution. (**a**) Data collection devices. (**b**) Distribution of reference data.

The data used in this study were collected by the RM-1290 X-band marine radar. This is an X-band short-pulse radar operating in HH polarization mode. The radar frequency is approximately 9.6 GHz, the horizontal beam width is no more than 1.3°, and the vertical beam width is 23° ± 2. The receiver has an intermediate frequency bandwidth of 20 ± 3 MHz. The radar antenna is installed at a height of approximately 40 m above the sea surface and transmits radio waves towards the sea surface at a low grazing angle. The received echo signals are Bragg scattered back from the sea surface. The radar images are collected at an interval of 2.5 s and acquired using a 14-bit digital acquisition card. The echo images obtained by each pulse of the radar form a radar image sequence, with each group containing 32 images. The range of echo intensity is from 0 to 8192 on the Plan Position Indicator (PPI). Finally, the single radar image has a radial resolution of 7.5 m and an azimuthal resolution of 0.1°. The number of azimuth lines is approximately 3600, and the number of radial points is approximately 600. The effective radial distance of the radar is 4.5 km, which can effectively filter out part of the white noise signals. The installation layout of the radar hardware and related sensor devices is shown in Figure 9a.

The experimental data, comprising 1283 sets, were collected at the shore base of Haitan Island in Fujian Province, China, during the period from October to December 2010. The data collection process was influenced by Typhoon ‘Megi’. Owing to heavy rainfall, 136 image sequences were eliminated by applying the preprocessing method described in Section 2, and 1147 sets of image sequences were retained for wind speed model testing.

The reference data were collected using a Model-05103 anemometer. Its wind speed measurement resolution is 0.1 m/s, the wind direction resolution is 1°, and the sampling period is 1 min. In order to make the radar sampling data consistent with the anemometer data, the anemometer data were sampled at the same frequency as the radar. After sampling, the reference wind direction ranged from 10° to 350°, and the reference wind speed range was from 1.4 m/s to 20.2 m/s. The reference wave parameters were sourced from the MIROS Wavex system, which was connected to the same radar hardware. The WAVEX system can provide detailed wave parameters, which can be used as references for different sea conditions to test the performance of the proposed method under various scenarios. Due to the repetitive data in ocean dynamic parameters during this period, after removing the duplicates, a total of 125 sets of wave height and current direction data were used as diverse references for different scenarios. The distribution range of wave height ranges between 0.03 m and 3.46 m, and the distribution range of current direction ranges from 64° to 257°, as shown in Figure 9b.

### 4.2. Results Validation

**Figure 10:**
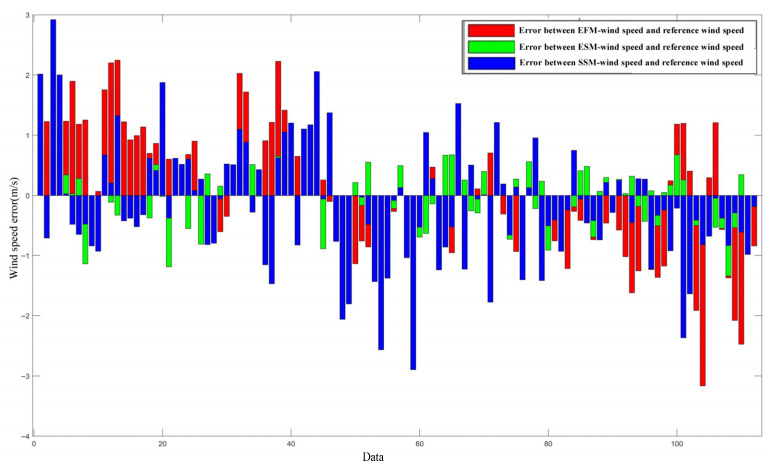
Comparison of errors between the retrieval results and the reference wind speed.

To evaluate the performance of the model proposed in this study, a series of experiments is conducted. The Empirical Function Model [21] is used as a reference for comparison, as described earlier. The wind speed retrieved by the energy stable value model is denoted as “ESM-wind speed”, that retrieved by the entropy stable value model is referred to as “SSM-wind speed”, and the one retrieved by the Empirical Function Model is called “EFM-wind speed”. Figure 10 shows the error distribution of the three models with respect to the reference wind speed. A total of 112 sets of data were extracted from the different sea state scenarios indicated by the WAVEX data.

For ESM wind speed, the error ranges from −2 to 1 m/s, and the error fluctuation is relatively small compared to the other two methods. Moreover, half of the data error points fall within the relatively narrow error ranges of −0.5 m/s to 0.5 m/s. For SSM wind speed, the error distribution ranges from −2 m/s to 2 m/s. Among them, more than 50% of the inversion results have error concentration ranges within −2 m/s to 1 m/s, and they show greater fluctuation compared to the ESM wind speed errors.

**Figure 11:**
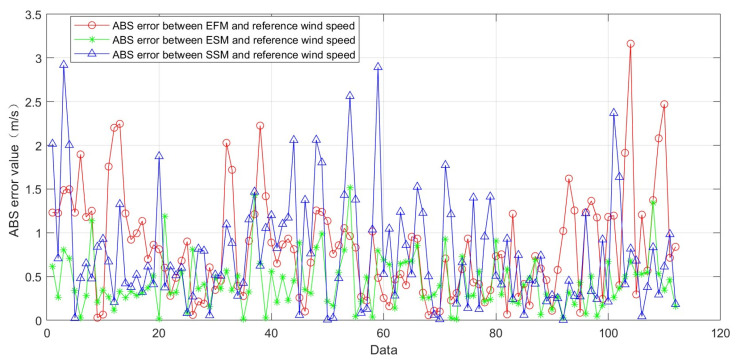
Comparison of absolute error value between the retrieval results and the reference wind speed.

The error distribution of the EFM wind speed and the reference wind speed remains within the range of −2 m/s to 2 m/s, which is similar to that of the SSM wind speed; however, approximately 65% of the errors lie between −1 and 2 m/s, and about 10% of the error points have absolute values exceeding 2 m/s. The correlation coefficient, average error, and root mean square error (RMSE) relative to the reference wind speed were obtained for these three models, as shown in Table 1. Taking into account the results in Figure 10 and Table 1, it can be observed that the wind speed retrieval results of the two models proposed in this paper are more accurate than the Empirical Function Model, and their error fluctuations and concentration ranges are smaller for the data currently used. Figure 11 presents a comparison of absolute error values between the retrieval results and the reference wind speed. EFM (red circle line) exhibits large fluctuations in absolute error, with high peaks and some points exceeding 3 m/s. ESM (green star line) shows relatively stable absolute errors, mostly within 1.5 m/s. SSM (blue triangle line) has a wide error distribution, with peak values close to those of EFM. These results indicate significant differences in wind speed prediction accuracy among the models, with ESM demonstrating relatively better stability, while EFM and SSM show larger error fluctuations. Statistical analysis yields average absolute errors of 0.84 m/s, 0.43 m/s, and 0.77 m/s for EFM, ESM, and SSM, respectively.

**Figure 12:**
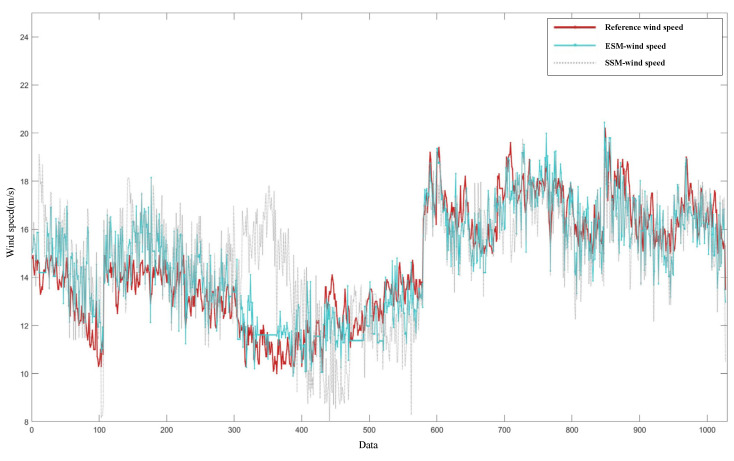
Comparison of the retrieval results of the energy and entropy stable value wind speed models with the reference wind speed.

Figure 12 presents the wind speed results derived from the three models for 1028 sets of data, along with the reference wind speed values. The red solid line represents the reference wind speed, the blue solid line represents the ESM wind speed, and the gray dotted line represents the SSM wind speed. In terms of the overall trend, the wind speed retrieved using the energy stable value (the blue solid line) is closer to the reference wind speed (the red solid line), and the correlation is stronger. For data points 300~400, which correspond to large-angle wind directions, the accuracy of both models decreases to some extent; however, compared with the entropy stable value model, the method of estimating wind speed using the energy stable value model maintains good stability. The change in wind direction angles leads to a redistribution of the wind speed profile.

Wind speed can change due to factors such as ocean surface temperature, resulting in a correlation between wind speed and wave height, which is known as the wind shear phenomenon. Under these conditions, the wind streaks in the marine radar image sequence also become more disordered, thus affecting the retrieval results for sea surface wind speed. The energy feature is computed on the basis of the sum of the squares of the elements in the GLCM. If the overall gray-level distribution pattern of the image texture remains unchanged, the energy value exhibits better noise resistance.

**Figure 13:**
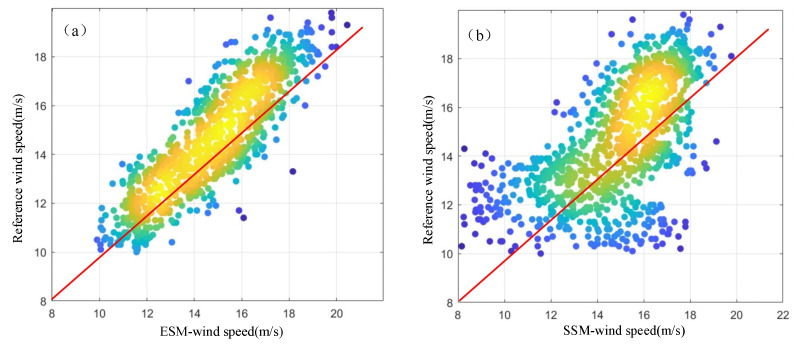
Correlation between retrieved wind speeds (energy and entropy models) and reference wind speed. (**a**) Energy stable model vs. reference. (**b**) Entropy stable model vs. reference.

Figure 13 depicts the correlation distribution of the wind speed retrieved by the energy and entropy stable value models compared to the reference wind speed. As shown in Figure 13a, the wind speed estimation results of the energy stable value model are clustered around either side of the red solid line. This indicates that the energy stable model has a strong correlation with the reference wind speed, and the retrieved results are relatively close to the measured values; however, in Figure 13b, the wind speed estimation results of the entropy stable value model are distributed more randomly, with a weak correlation with the reference wind speed. The statistical data reveal that the correlation coefficient for the energy stable value model is 0.89, with an RMSE of 2.1 m/s. For the entropy stable value model, these values are 0.72 and 2.2 m/s, respectively.

**Figure 14:**
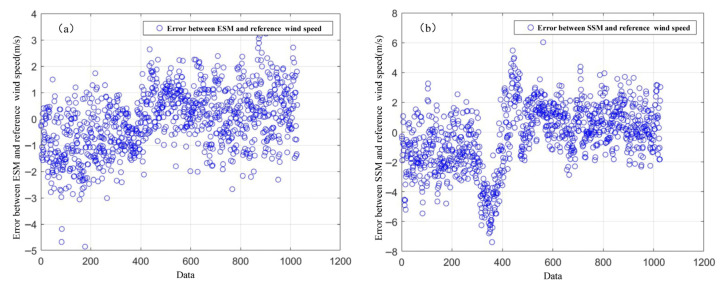
Distribution of errors between the retrieved wind speed and the reference wind speed (**a**) The error between ESM and reference wind speed (**b**) The error between SSM and reference wind speed.

Figure 14 illustrates the error distribution of retrieved sea surface wind speeds from the energy and entropy stable value models compared to the reference wind speeds for all 1028 datasets. Figure 14a shows that the errors from the energy stable value model are mainly concentrated between −3 and 2 m/s. Notably, about 59% of these errors fall within the narrow range of −1 to 1 m/s. This indicates that the energy stable value model can estimate wind speed fairly accurately in most cases, as its errors are tightly clustered, demonstrating good stability and accuracy. In contrast, Figure 14b shows that the error distribution for the entropy stable value model is much wider (roughly distributed between −6 and 4 m/s), and only 36% of its errors lie within −1 to 1 m/s. This suggests that the entropy stable value model is comparatively less stable, and its precision is inferior to that of the energy stable value model. Overall, these results confirm that the energy stable value model offers greater accuracy and more robust performance for wind speed retrieval.

### 4.3. Model Applicability Verification

**Figure 15:**
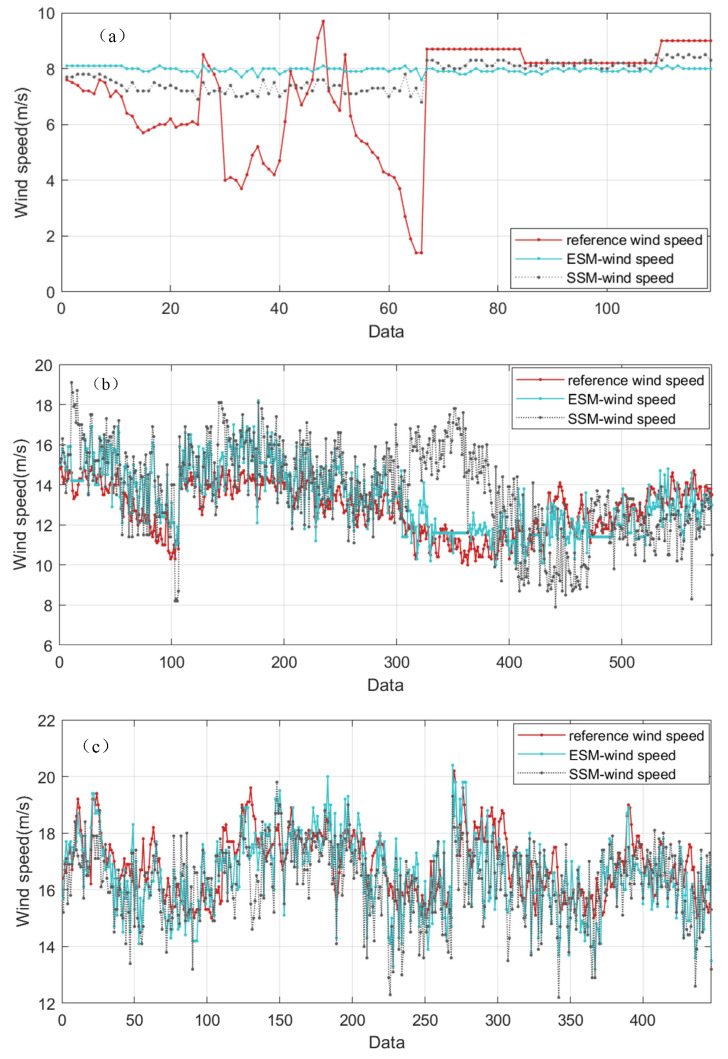
Wind speed retrieval results of the two models compared with the reference wind speed. (**a**) Low wind speed. (**b**) Moderate wind speed. (**c**) High wind speed.

To evaluate the performance of the two models under different wind speed ranges, we categorized the dataset into 119 low wind speed cases (1.4–10 m/s), 581 moderate wind speed cases (10–15 m/s), and 447 high wind speed cases (15–20.2 m/s). The retrieval results of each model were then compared to the reference wind speed. The results are shown in Figure 15, and the error statistics are presented in Table 2.

Figure 15a shows that at low wind speeds, both models have relatively low retrieval accuracy. In particular, for speeds less than 6 m/s (see data points at 30–40 and 55–65), neither model exhibits good tracking ability, and both fail to accurately estimate the wind speed information. This is because at low wind speeds, the texture features of small-scale wind streaks in the radar sequences are relatively weak, and the inter-image correlation is poor. Consequently, the performance of extracting wind speed information from the gray-level co-occurrence matrix (GLCM) deteriorates. Poor image correlation increases the randomness of the texture, dispersing the probability distribution of different gray-level combinations. As a result, entropy remains relatively high while the contrast of the energy feature becomes more pronounced, suggesting that an energy-based inversion approach may still be effective under these conditions.

Once the wind speed exceeds 10 m/s (Figure 15b,c), both models exhibit acceptable tracking performance. When distinct wind streak textures appear in the images, both models are viable for wind speed retrieval. At moderate wind speed (10 to 15 m/s; Figure 15b), the wind speeds retrieved by employing the energy stable value model (blue solid line) follow the reference wind speed (the red solid line) more closely, and the two curves show a strong correlation. This holds true even under large wind direction changes (wind direction > 180°; data points 300–400 in Figure 15b). Although such a change in wind direction can cause wind shear and yield an inhomogeneous wind speed profile that reduces the retrieval accuracy, the energy stable value method still maintains relatively good stability.

The error statistics further show that for moderate winds, the errors of the energy stable value model are mainly between −3 m/s and 2 m/s, with approximately 56% falling in the narrow band of −1 m/s to 1 m/s. In contrast, the entropy stable value model produces a greater error distribution, with errors ranging between −6 m/s and 4 m/s, and only 26% of the errors fall within the interval of −1 m/s to 1 m/s. From Table 2, we can infer that at moderate wind speeds, the energy stable value model is superior to the entropy stable value model in both accuracy and stability.

At high wind speeds (15–20.2 m/s; Figure 15c), both models demonstrate excellent stability. As shown in Table 2, the accuracy of both models under high winds is better than at low and moderate wind speeds. At higher wind speeds, the small-scale wind streak features become more conspicuous, and the values of these streak features more directly reflect variations in wind speed. For high wind speeds, the error distributions of each model were statistically analyzed, and the errors of the two models were compared to the reference wind speed. The ESM wind speed errors range from −2 m/s to 2 m/s, with 54% of the error points clustered between −1 m/s and 1 m/s. In comparison, the SSM wind speed errors range from −2 m/s to 4 m/s, with an error span of 6 m/s, and approximately 48% of the error points are distributed within the interval −1 m/s to 1 m/s. Table 2 indicates that the mean bias of the energy stable value model is about 48% lower than that of the entropy stable value model, and the RMSE is about 23% lower.

### 4.4. Verification of Different Sea Conditions

**Figure 16:**
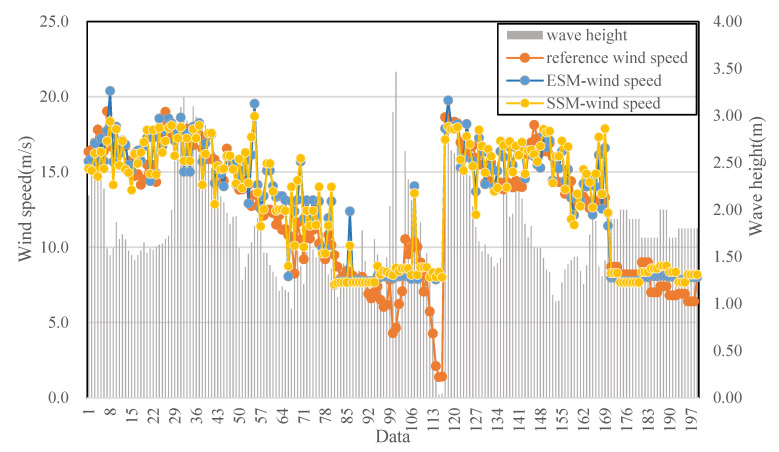
Wind speed retrieval results of two models compared with the reference wind speed under different wave height conditions.

Significant wave height can reflect the complexity of sea conditions and the distribution of ocean energy. Two sea conditions were defined as low sea state (wave height ranging from 0 m to 1.5 m) and high sea state (wave height ranging from 1.5 m to 3.46 m). The experiment tested the adaptability of the model to different sea conditions using different wave height information. A total of 199 sets of radar image sequences were selected with wave heights provided by the Wavex system data. The results under different wave height conditions are shown in Figure 16.

Table 3 illustrates the performance of the two wind speed retrieval models under varying sea conditions. The correlation coefficients of the ESM and SSM with reference wind speeds are 0.92 and 0.89, respectively. The mean deviations for the two models are 0.65 m/s and 0.61 m/s, while the RMSE values are 1.66 m/s and 1.81 m/s. Focusing on low sea states, the performance trends of the two models are reversed compared to their overall evaluation. The SSM demonstrates higher precision than the ESM, with both the mean deviation (0.30 m/s) and RMSE (0.81 m/s) being well below 1 m/s, aligning with the requirements of engineering applications. Conversely, under high sea states, the ESM achieves superior accuracy, with a correlation coefficient of 0.94, a mean deviation of 0.16 m/s, and an RMSE of 0.73 m/s, showcasing its robustness under more challenging conditions. These results highlight the models’ complementary strengths: SSM excels under calm sea conditions, while ESM is better suited for high sea states, offering practical insights for optimizing wind speed retrieval in diverse marine environments.

Using experiments with measured data, we evaluated both wind speed retrieval models under various wind and sea state conditions. Although both wind speed models can retrieve wind speed information, the entropy stable value model shows better accuracy and applicability when the speeds are below 10 m/s and the sea state is low. In contrast, the energy stable value model performs more accurately when wind speeds exceed 10 m/s and the sea state is high. For practical engineering applications, it is advisable to employ both models and to test them under a wide range of conditions.

## 5. Conclusions

This study proposes a novel sea surface wind speed modeling method based on X-band marine radar image sequences. Compared with traditional approaches that directly apply radar backscatter intensity wind speed models, the use of gray-level co-occurrence matrix (GLCM) features provides a more noise-resistant, pattern-recognition-capable, and structurally informative means of capturing the stable texture characteristics of small-scale wind streaks. By combining the stable texture features of small-scale wind streaks (energy stable value and entropy stable value) and performing piecewise fitting with wind speed measurements from a Model-05103 anemometer, a dual-parameter model for local wind speed retrieval is achieved. A three-stage preprocessing (noise suppression, geometric correction, and interpolation) is used to ensure image quality and enable effective extraction of small-scale wind streaks in Cartesian coordinates. The study systematically analyzes the relationship between gray-level co-occurrence matrix (GLCM) texture features from radar image sequences and measured wind speeds, confirming that the GLCM-derived energy feature is positively correlated with wind speed, whereas the entropy feature is negatively correlated with wind speed. Additionally, energy and entropy still retain a relatively consistent relationship with the sea surface wind speed when the wind speed is lower than 10 m/s.

Model validation using real data from an RM-1290 marine radar on the East China Sea coast, along with co-located wind instrument measurements, showed that both the energy-stable and entropy-stable wind speed models outperform the empirical function model in local tests. The average retrieval error was reduced by 49.3% and 16.7%, and the root mean square error by 48.5% and 4%, respectively. The energy-stable feature model demonstrated better retrieval accuracy and noise resistance. A subsequent evaluation with a large dataset compared the retrieval accuracy, wind speed applicability, and robustness under different sea states of the two models. The results indicate that overall, the energy stable value wind speed model is superior to the entropy stable value model, with correlation coefficients of 0.89 vs. 0.72 relative to the reference wind speed. Under moderate and high wind conditions, the energy stable value model’s retrieval accuracy and robustness are better than those of the entropy model. Notably, for wind speeds below 10 m/s, the entropy stable value model’s average deviation is 27.3% lower than that of the energy model, indicating an advantage at low wind speeds.

Studies under low and high sea states demonstrate the complementary strengths of the two models: the entropy stable value model excels in low sea states, while the energy stable value model is more suitable for high sea states. This provides a theoretical basis for optimizing marine radar wind speed modeling under different ocean conditions. These findings provide valuable insights for practical engineering applications, though further testing with expanded data and a broader data acquisition range (e.g., different radar systems and oceanic conditions), particularly for wind speeds below 6 m/s, is needed to enhance model reliability. As this study focuses on the core characteristics and patterns of sea surface wind speed errors under rain-free conditions, rain data were initially excluded to avoid interference of this complex factor with the fundamental patterns. Future work will include an in-depth investigation into the impact of rainfall on sea surface wind speed retrieval models.

## Figures and Tables

**Table 1:** Comparison of three wind speed models’ results with reference wind speed.

Model	Correlation Coefficient	Average error (m/s)	Root Mean Square Error (m/s)
Empirical function model	0.83	1.50	1.03
Energy stable value model	0.96	0.76	0.53
Entropy stable value model	0.84	1.25	0.99

**Table 2:** Error statistics for wind speed retrieval under different wind speeds.

Wind Speed	Performance	ESM	SSM
Low wind speed	correlation coefficient	0.76	0.77
	average deviation (m/s)	0.88	0.64
	RMSE (m/s)	1.83	1.5
Moderate wind speed	correlation coefficient	0.88	0.81
	average deviation (m/s)	−0.39	−0.94
	RMSE (m/s)	1.21	2.54
High wind speed	correlation coefficient	0.92	0.83
	average deviation (m/s)	0.29	0.56
	RMSE (m/s)	1.08	1.41

**Table 3:** Comparison of wind speed retrieval results under different wave height conditions.

Sea Conditions	Wind Speed Model	Correlation Coefficient	Mean Deviation (m/s)	RMSE (m/s)
Low sea state	Energy stable value model	0.81	0.64	1.16
	Entropy stable value model	0.89	0.30	0.81
High sea state	Energy stable value model	0.94	0.16	0.73
	Entropy stable value model	0.82	0.17	1.18

## Data Availability

The data that support the findings of this study are available from the corresponding author upon reasonable request.
